# Arenavirus Induced CCL5 Expression Causes NK Cell-Mediated Melanoma Regression

**DOI:** 10.3389/fimmu.2020.01849

**Published:** 2020-08-21

**Authors:** Hilal Bhat, Gregor Zaun, Thamer A. Hamdan, Judith Lang, Tom Adomati, Rosa Schmitz, Sarah-Kim Friedrich, Michael Bergerhausen, Lamin B. Cham, Fanghui Li, Murtaza Ali, Fan Zhou, Vishal Khairnar, Vikas Duhan, Tim Brandenburg, Yara Maria Machlah, Maximilian Schiller, Arshia Berry, Haifeng Xu, Jörg Vollmer, Dieter Häussinger, Beatrice Thier, Aleksandra A. Pandyra, Dirk Schadendorf, Annette Paschen, Martin Schuler, Philipp A. Lang, Karl S. Lang

**Affiliations:** ^1^Medical Faculty, Institute of Immunology, University Duisburg-Essen, Essen, Germany; ^2^Department of Medical Oncology, West German Cancer Center, University Hospital Essen, University Duisburg-Essen, Essen, Germany; ^3^Department of Systems Biology, Beckman Research Institute, City of Hope, Monrovia, CA, United States; ^4^Department of Molecular Medicine II, Medical Faculty, Heinrich Heine University, Düsseldorf, Germany; ^5^Abalos Therapeutics GmbH, Essen, Germany; ^6^Department of Gastroenterology, Hepatology and Infectious Diseases, University of Düsseldorf, Düsseldorf, Germany; ^7^Department of Dermatology, University Hospital Essen, Essen, Germany; ^8^German Cancer Consortium (DKTK), Partner Site University Hospital Essen, Essen, Germany

**Keywords:** arenavirus, virotherapy, immunotherapy, LCMV, innate immunity, CCL5, NK cells, melanoma

## Abstract

Immune activation within the tumor microenvironment is one promising approach to induce tumor regression. Certain viruses including oncolytic viruses such as the herpes simplex virus (HSV) and non-oncolytic viruses such as the lymphocytic choriomeningitis virus (LCMV) are potent tools to induce tumor-specific immune activation. However, not all tumor types respond to viro- and/or immunotherapy and mechanisms accounting for such differences remain to be defined. In our current investigation, we used the non-cytopathic LCMV in different human melanoma models and found that melanoma cell lines produced high levels of CCL5 in response to immunotherapy. *In vivo*, robust CCL5 production in LCMV infected Ma-Mel-86a tumor bearing mice led to recruitment of NK cells and fast tumor regression. Lack of NK cells or CCL5 abolished the anti-tumoral effects of immunotherapy. In conclusion, we identified CCL5 and NK cell-mediated cytotoxicity as new factors influencing melanoma regression during virotherapy.

## Introduction

Melanoma accounts for the great majority of skin cancer related deaths. However, over the past few years, immunotherapy has dramatically changed the landscape of melanoma treatment. The immune system can directly attack tumor cells via tumor antigen-specific cytotoxic CD8^+^ T cells, activated natural killer (NK) cells or antibody-mediated cytotoxicity (1). CD8^+^ T cells are considered to be the main anti-tumor effectors and their contribution to tumor regression has been shown to be of relevance in patients with melanoma and lung carcinoma (2, 3). In contrast to CD8^+^ T cells, the role of NK cells in tumor regression has not been as thoroughly studied. Interestingly, there are some studies demonstrating that NK cells play critical roles in reducing lung metastases in mouse models (4–6). NK cells are not frequently detected within tumor biopsies (7), and in melanoma, high levels of NK cell infiltrates generally correlate with strong tumor regression (7, 8). Once NK cells are recruited into the tumor microenvironment, NK activating NKp46, NKp30, NKp44, NKG2D and inhibiting KIR, NKG2A receptors can recognize and be activated by tumor cells (9, 10), leading to NK cell mediated cytotoxicity. Thus, NK cells might play a role in eliminating melanoma cells that are refractory to CD8^+^ T cell recognition through down-regulation of antigen presenting machinery and other escape mechanisms (11–13). Activated NK cells kill the target cells with cytolytic granules including perforin, granzyme (A and B), and granulysin (14). Despite the strong potential of NK cells to lyse tumor cells, NK cells barely infiltrate into solid tumors (15–18), indicating that new strategies to attract NK cells into the tumor microenvironment need to be developed.

The chemokine (C-C motif) ligand 5 (CCL5; RANTES) is crucial for maintaining several functions of T cells such as survival (19), migration (20), and differentiation (21). In the absence of CCL5, CD8^+^ T cells undergo enhanced exhaustion which can exacerbates viral infections and decrease virus specific CD8^+^ T cells (22). CCL5 is expressed in a wide variety of cell types including immune cells. CCL5 is also a potent chemoattractant for many cell types including monocytes, NK cells (23), memory T cells (24), eosinophils (25), and dendritic cells (26, 27). In melanoma, NK cell infiltration was shown to be driven by secretion of high levels of CCL5 (7). How CCL5 production is induced in tumor tissues and whether it can be modulated therapeutically remains to be elucidated. CCL5 expression has been detected in several tumor types including ovarian (28), prostate (29), pancreatic (30), and melanoma cancer (31). It therefore represents an important chemokine in the field of cancer biology which warrants further investigation.

The arenavirus lymphocytic choriomeningitis virus (LCMV) is a non-cytopathic virus with immune mediated anti-tumoral effects (32). Additionally, LCMV is known to activate NK cell-mediated cytotoxicity (33–35). Using an *in vivo* model of human melanoma, we found that LCMV treatment resulted in strong CCL5 production, NK cell infiltration and CCL5-dependent immune-mediated melanoma regression.

## Results

### Human Ma-Mel-86a Melanoma Cell Line Produces CCL5 Following LCMV Infection

**Figure 1:**
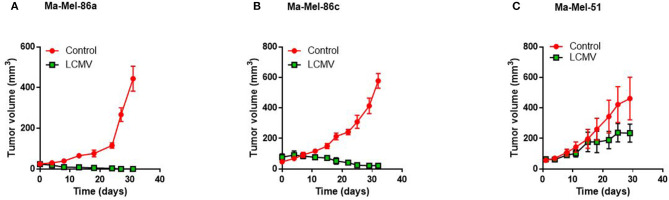
Human Ma-Mel-86a melanoma cell line produces CCL5 after LCMV infection. NOD/SCID mice were injected with 2 × 10^6^ melanoma cells subcutaneously in the left flank and once the tumor diameter was about 5 mm, mice were infected with 2 × 10^6^ PFU LCMV WE intratumorally or left untreated. Tumor growth of Ma-Mel-86a (**A**; *n* = 4–5), Ma-Mel-86c (**B**; *n* = 3), and Ma-Mel-51 (**C**; *n* = 4) was monitored at the indicated time points.

**Figure 2:**
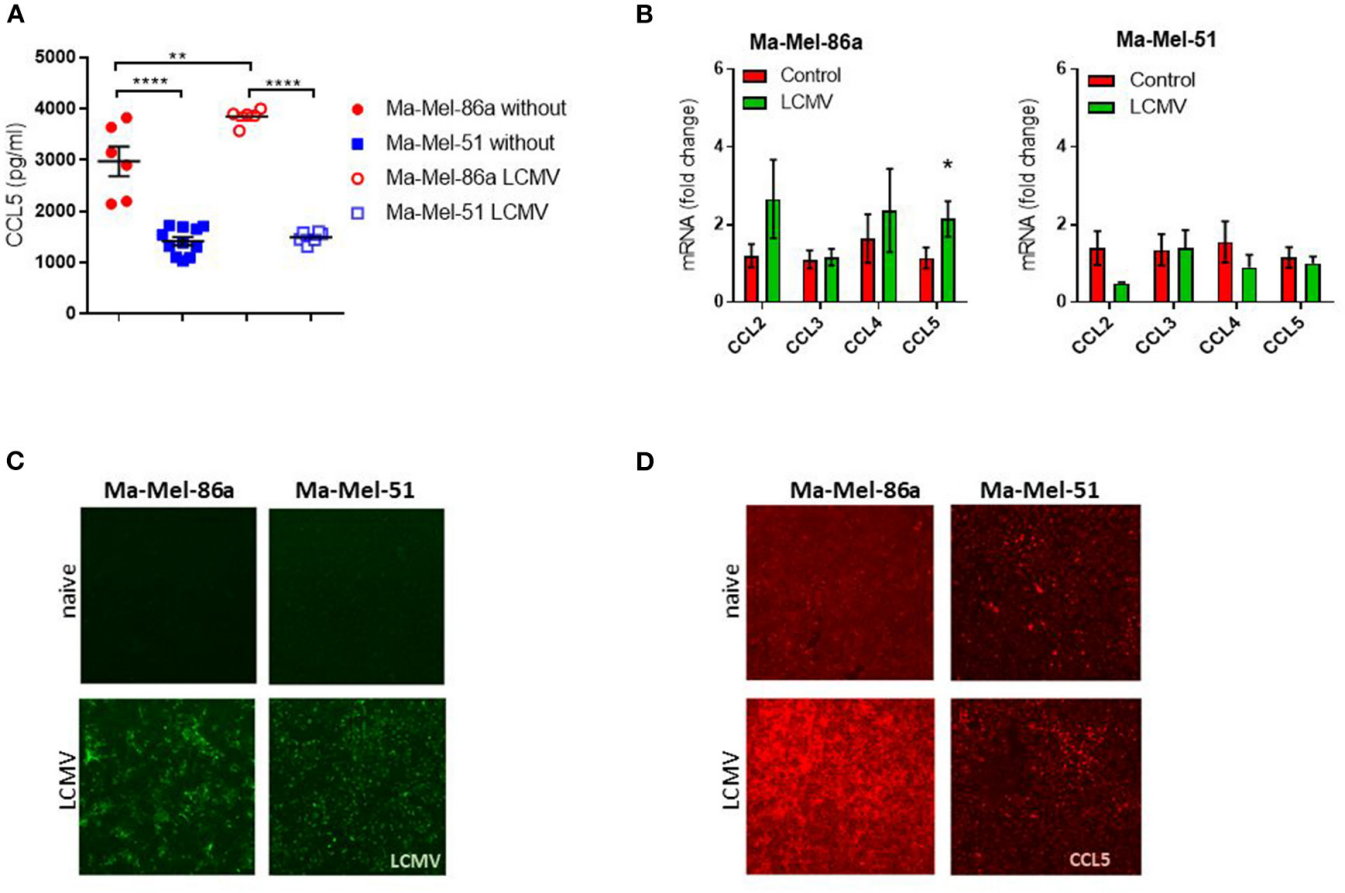
Human Ma-Mel-86a melanoma cell line produces CCL5 after LCMV infection. Ma-Mel-51 and Ma-Mel-86a cells were cultured in a 24 well plate at a density of 2 × 10^5^ cells/well and infected with LCMV WE (multiplicity of infection [MOI] 1). **(A)** Expression of CCL5 in the culture supernatant was assessed by ELISA (*n* = 6). **(B)** Expression of LCMV nucleoprotein (green) was stained and analyzed by fluorescent microscopy (*n* = 4). **(C)** Expression of different chemokines was checked by qRT-PCR (*n* = 6). **(D)** Expression of CCL5 (red) was stained and analyzed by fluorescent microscopy (*n* = 3). Data are shown as mean ± s.e.m. Significant differences between the two groups were detected by unpaired two-tailed *t*-tests and are indicated as follows: ns, not significant; *p < 0.05; **p < 0.01; ***p < 0.001; ****p < 0.0001.

To determine immunological signals influencing immunotherapy, we first tested the response of different melanoma cell lines to LCMV treatment *in vivo*. Comparing the three human melanoma cell lines in a murine xenograft model, Ma-Mel-86c Ma-Mel-86a and Ma-Mel-51 (36, 37), we found that LCMV infection strongly suppressed growth of Ma-Mel-86a tumors (Figure 1A) and regressed tumor outgrowth in case of Ma-Mel-86c (Figure 1B). However, LCMV infection did not lead to a significant reduction in Ma-Mel-51 tumor growth (Figure 1C). The strong anti-tumoral effects observed with the Ma-Mel-86a cells were recapitulated by intratumoral and systemic LCMV application (Supplementary Figure 1). However, neither intratumoral nor intravenous application of LCMV led to a significant reduction in Ma-Mel-51 tumor bearing mice. We hypothesized that a differential chemokine expression profile might be responsible for the observed differences in treatment efficiency between these cell lines. CCL5 is one important chemokine, which has been described to enhance tumor formation and progression, but also to have important immune activating features in melanoma (31). Furthermore, expression of CCL5 can be induced by Type I Interferon (IFN-I) (38), which is highly induced following LCMV infection (39). We therefore wondered whether CCL5 expression would change in our model upon LCMV-WE infection. *In vitro*, we detected enhanced CCL5 protein expression in Ma-Mel-86a cells when compared to Ma-Mel-51 cells (Figure 2A). Upon LCMV infection, LCMV propagated similar in Ma-Mel-86a and Ma-Mel-51 cells (Figure 2B), suggesting that there is difference between these cell lines which may affect virus infection, replication or budding. Infection with LCMV further increased CCL5 in Ma-Mel-86a cells, but not in Ma-Mel-51 cells (Figure 2A). RT-PCR and immunofluorescence further confirmed the increased CCL5 expression in Ma-Mel-86a cells following LCMV infection (Figures 2C,D). No substantial differences in type I interferon (IFN-I) responses and expression of interferon stimulated genes (ISGs) were observed between Ma-Mel-86a, Ma-Mel-51, and other melanoma cell lines *in vitro* (Supplementary Figure 2).

### CCL5 Is Produced in Ma-Mel-86a Tumors *in vivo* Upon Infection With LCMV

**Figure 3:**
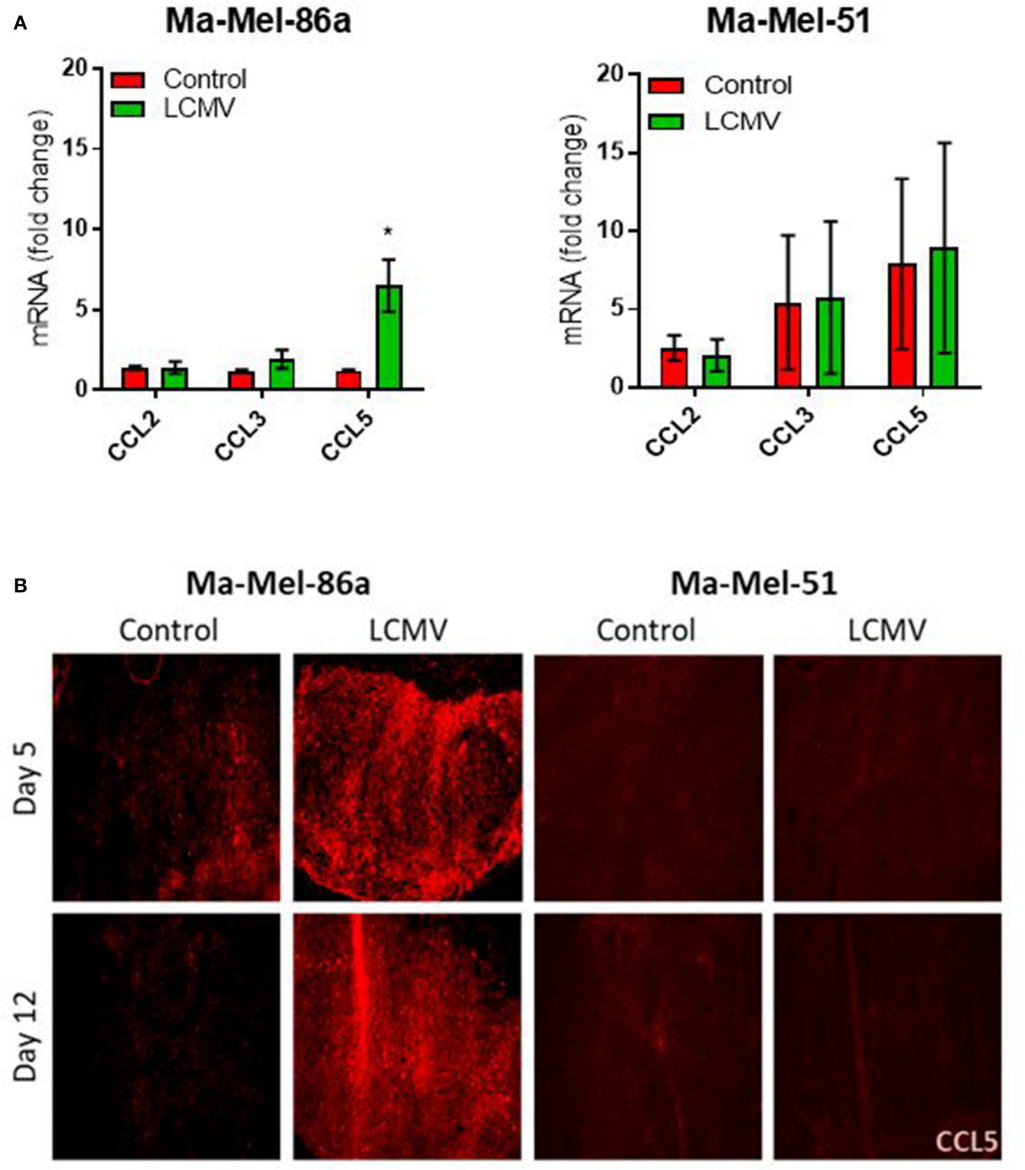
CCL5 is produced in Ma-Mel-86a derived tumors *in vivo* upon infection with LCMV. NOD/SCID mice were injected with 2 × 10^6^ Ma-Mel-86a or Ma-Mel-51 cells subcutaneously in the left flank and once the tumor diameter was about 5 mm, they were infected with 2 × 10^6^ PFU LCMV WE intratumorally or left untreated. qRT-PCR for chemokine expression in the tumor was analyzed on day 10 (**A**; *n* = 4). The expression data for each gene and cell line was normalized to the corresponding control measurement. Mice were sacrificed on day 5 and day 12 after LCMV intratumoral injection and the expression of CCL5 (red) was analyzed by immunofluorescence (**B**; *n* = 4). Data are shown as mean ± s.e.m. and analyzed by unpaired Student's *t*-test. *P < 0.05.

Next, we investigated whether LCMV therapy also induced CCL5 production in Ma-Mel-86a tumors *in vivo*. Intratumoral LCMV administration in Ma-Mel-86a tumors established in NOD/SCID mice led to a significant upregulation of CCL5 at the transcriptional level, while in Ma-Mel-51 tumor bearing mice the CCL5 mRNA levels remained unchanged (Figure 3A). We confirmed this data using immunofluorescence (Figure 3B). Murine and human CCL5 proteins show differences in homology between species (40), and RT-PCR primers and primary antibodies used to detect human CCL5 do not cross react with murine CCL5. Therefore, these experiments clearly demonstrate that CCL5 was produced intrinsically by the xenografted human melanoma cells. We concluded that the strong anti-tumoral effects of LCMV in Ma-Mel-86a melanoma cells correlated with enhanced CCL5 production *in vivo*.

### CCL5 Is Functionally Important for Antitumoral Activity

**Figure 4:**
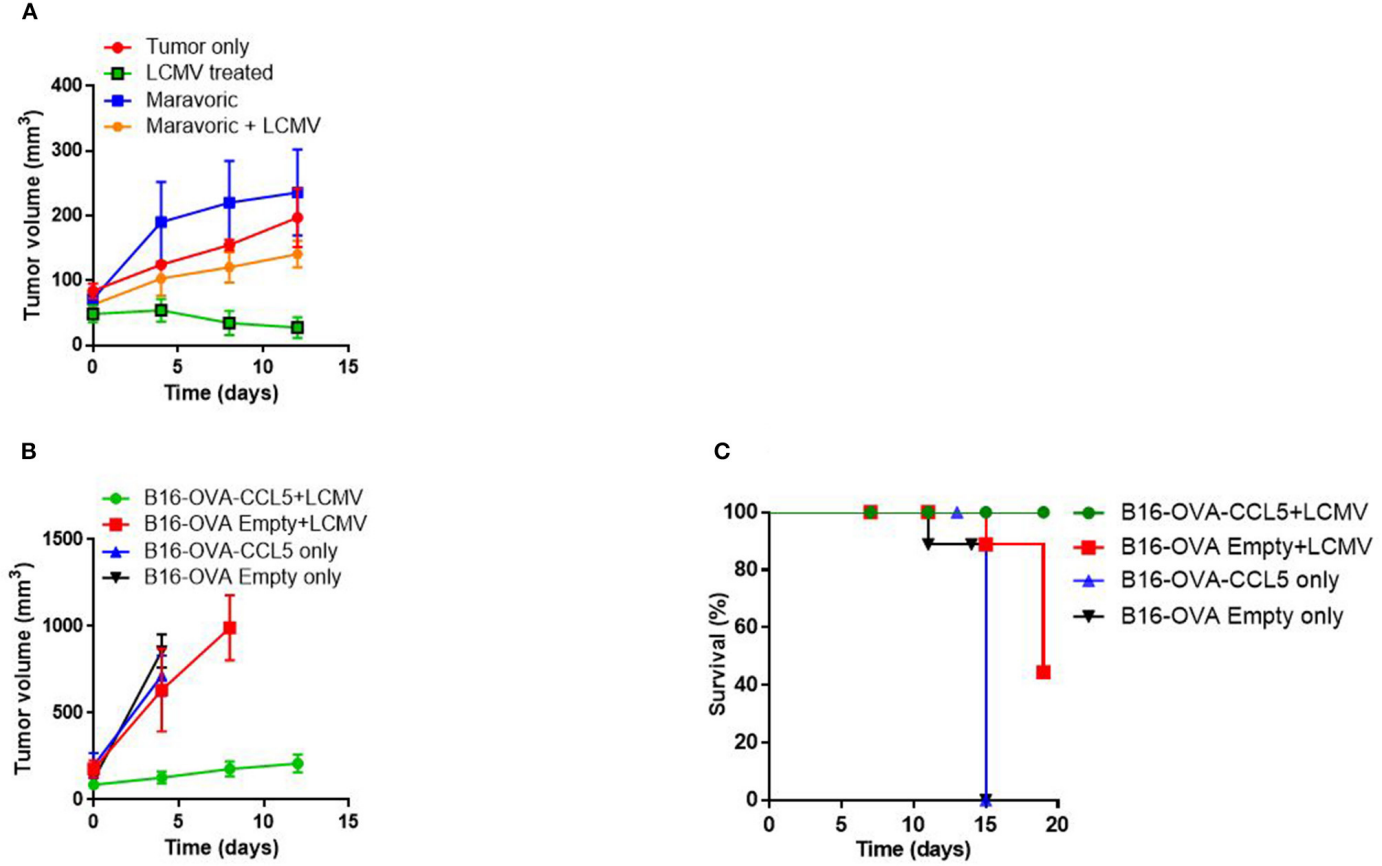
CCL5 is important for anti-tumoral activity. **(A)** 2 × 10^6^ Ma-Mel-86a melanoma cells were injected subcutaneously in the flank of NOD/SCID mice. One group was treated with Maraviroc (50 mg/kg body weight/mice), another with the combination of Maraviroc and 2 × 10^6^ PFU LCMV WE, the third group treated with LCMV only and the last group was left untreated. Tumor growth was followed as indicated (*n* = 3). **(B,C)** 2 × 10^6^ B16-Ova empty vector or B16-Ova-CCL5 cells, overexpressing CCL5, were injected subcutaneously in the flank of C57B6 mice. Once the tumor diameter was about 5 mm, they were infected with 2 × 10^6^ PFU LCMV WE intratumorally or left untreated. Tumor growth **(B)** and survival **(C)** were monitored (*n* = 4).

Next we wondered whether CCL5 production is functionally linked to the observed anti-tumoral activity following LCMV treatment. Therefore, we blocked CCL5 with Maraviroc, a CCR5 inhibitor used for the treatment of human immunodeficiency virus (HIV) infection. Mechanistically, Maraviroc binds to the transmembrane pocket of CCR5 and is a slow-offset functional antagonist that prevents internalization (41, 42). We treated tumor-bearing NOD/SCID mice with Maraviroc and subsequently infected them with LCMV. Administration of Maraviroc led to a slightly increased tumor volume and combination therapy with LCMV diminished LCMV's anti-tumoral activity whereas LCMV treatment alone significantly decreased tumor growth (Figure 4A). To molecularly corroborate the phenotype, we made use of the B16-Ova murine melanoma cell line overexpressing CCL5 (B16-Ova-CCL5) and compared them to control cells (B16-Ova-Empty) following LCMV therapy. We found that overexpression of CCL5 as well as LCMV treatment alone resulted in slightly decreased tumor growth and extended survival as in LCMV only treated mice (Figures 4B,C). We speculate that LCMV treatment alone is potentially not sufficient to upregulate CCL5 expression in the B16 syngeneic melanoma model. However, the combination of CCL5 overexpression and LCMV treatment was highly effective in restricting the tumor and increasing survival of mice (Figures 4B,C). These findings further suggest that CCL5 is a critical factor for anti-tumoral activity induced by LCMV. However, additional and yet unknown parameters might be altered by LCMV.

### CCL5 Induces NK Cell Mediated Cytotoxicity

**Figure 5:**
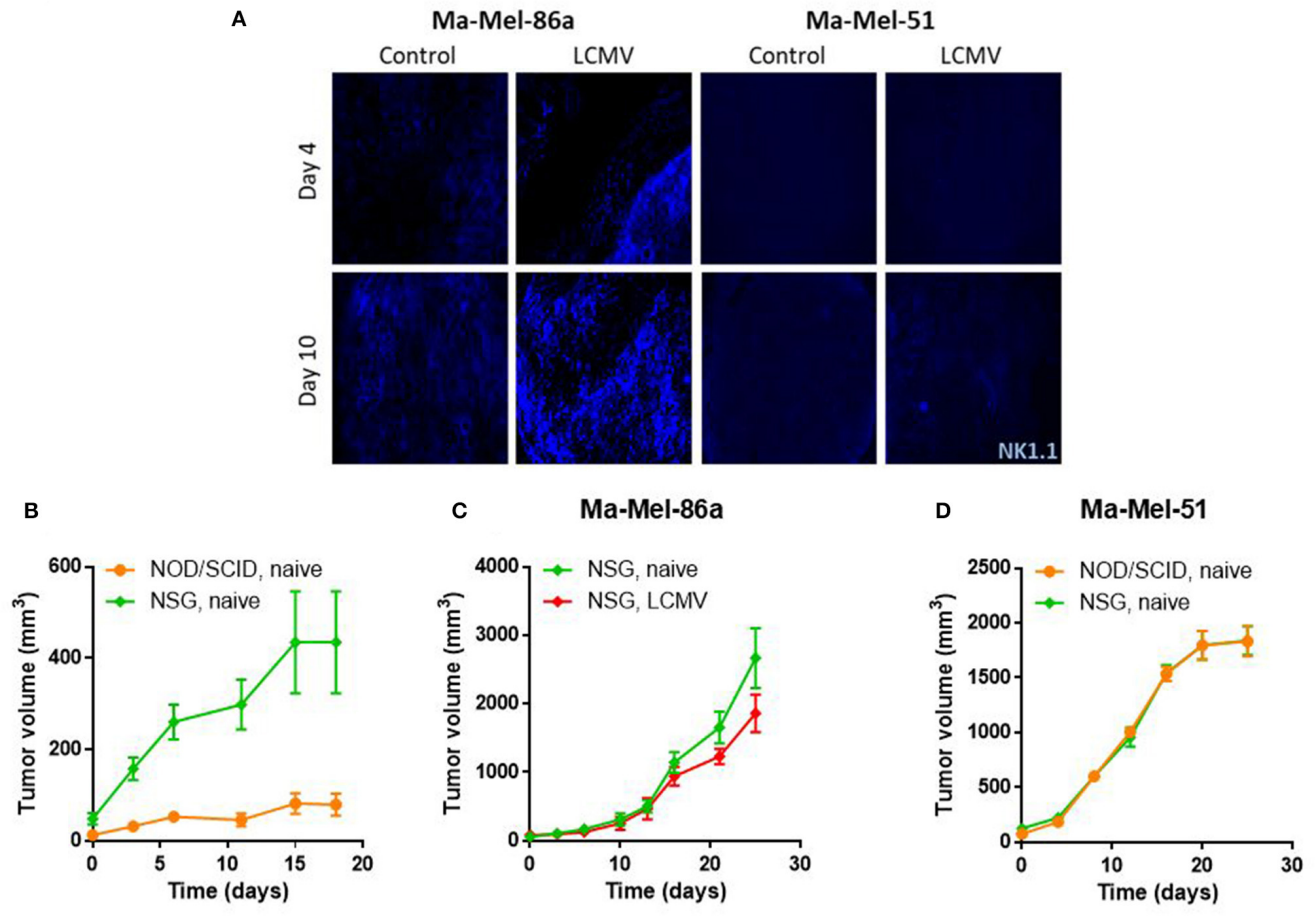
CCL5 induces NK cell mediated cytotoxicity. **(A)** NOD/SCID mice were injected with 2 × 10^6^ Ma-Mel-86a or Ma-Mel-51 cells subcutaneously in the left flank. Once the tumor diameter was about 5 mm, they were infected with 2 × 10^6^ PFU LCMV WE intratumorally or left untreated. Mice were sacrificed on day 4 and day 10 after LCMV WE injection and infiltration of NK cells (blue) was analyzed by immunofluorescence (*n* = 4). **(B)** NOD/SCID (*n* = 4) or NSG (*n* = 5) mice were injected with 2 × 10^6^ Ma-Mel-86a cells subcutaneously in the left flank. Tumor growth was followed. **(C)** NSG mice were injected with 2 × 10^6^ Ma-Mel-86a cells subcutaneously in the left flank and once the tumor diameter was about 5 mm, they were treated with 2 × 10^6^ PFU LCMV WE intratumorally or left untreated. Tumor growth was followed (*n* = 4). **(D)** NOD/SCID and NSG mice were injected with 2 × 10^6^ Ma-Mel-51 cells subcutaneously in the left flank and left untreated. Tumor growth was followed (*n* = 4).

NOD/SCID mice lack a adaptive as well as some innate immune responses but do have NK cells. Previously, it was shown in a melanoma model that NK cell infiltration into the tumor is mediated via CCL5 (7). We therefore wanted to investigate the NK cell dependent cytotoxicity in our tumor model. Indeed Ma-Mel-86a tumors showed strong NK cell infiltrates during LCMV immunotherapy (Figure 5A). In contrast, Ma-Mel-51 tumors demonstrated only limited NK cell recruitment (Figure 5A). Next, we treated NOD/SCID gamma (NSG) mice, which lack NK cells, with LCMV. Without infection, Ma-Mel-86a melanomas grew considerably faster in NSG mice when compared to NOD/SCID mice (Figure 5B). This indicates that in the absence of LCMV infection, NK cell-mediated cytotoxicity plays an important role during Ma-Mel-86a melanoma growth. When Ma-Mel-86a bearing NSG mice were infected with LCMV, LCMV did not demonstrate anti-tumoral effects (Figure 5C). This suggests that in the absence of NK cells, LCMV was not effective as a viro-therapeutic agent. Next we analyzed the role of NK cells on tumor regression in Ma-Mel-51 melanomas. In line with the CCL5 expression, Ma-Mel-51 melanomas grew similarly in NSG mice compared to NOD/SCID mice (Figure 5D), suggesting that NK cells do not affect Ma-Mel-51 growth. Taken together, we conclude that NK cells are recruited into the tumor tissue after LCMV mediated tumoral expression of CCL5 and that the NK cells exert strong anti-tumoral effects in the Ma-Mel-86a melanoma cells. Administration of LCMV was effective in Ma-Mel-86a tumors following intratumoral as well as intravenous injection (Supplementary Figure 1), while no substantial effect on Ma-Mel-51 tumors could be observed.

## Discussion

Talimogene laherparepvec (T-VEC), is a herpes simplex based oncolytic virus that was recently approved for the treatment of advanced melanoma (43). While complete response rates were found to be relatively low, at around 10.8% (44), combination therapy with PD-1 for instance, appears to increase this rate to around 33% (45). However, a disadvantage of T-VEC is that it has to be applied several times intra-tumorally into each metastasis (44). There is a high demand for novel immuno-therapeutic approaches to treat melanoma. In our study we examined the effect of the non-oncolytic arenavirus LCMV in a mouse melanoma model. Recently, it was shown that treatment of several syngeneic or spontaneous murine and human xenograft tumor models with LCMV resulted in regression (29) or complete elimination of tumors (32). LCMV preferentially replicated in tumor cells and metastatic sites leading to robust immune infiltration. Importantly, IFN-I did not interfere with LCMV replication within a tumor allowing for sustained innate immune activation and clearance of LCMV from other organs. There is a heterogenous response within and between tumor tapes in terms of response to LCMV therapy. In the present study we identified CCL5 production as one important factor determining the efficacy of treatment of melanoma with LCMV. However, it should be mentioned that in other studies, using breast cancer models, CCL5 was shown to exert an opposing role (7, 46). Therefore, we cannot draw any conclusions on the role of CCL5 in other non-melanoma tumors.

A strong positive correlation between the expression of CCL5 and the infiltration of NK cells into human melanoma biopsies as well as various other solid tumors (47) was previously demonstrated. Survival of patients with melanoma with a high expression of CCL5 was increased (7, 8). Additionally, infection with arenaviruses activates NK cell mediated cytotoxicity (15, 16), and NK cell deficiency promotes tumor outgrowth, whereas upregulation of NK cells inhibits tumor growth in different models (7, 48). Melanomas are especially very sensitive to NK cell mediated cytotoxicity (7, 49). Here we observed that LCMV replication in tumor cells induces CCL5 production, which in turn leads to a strong and robust anti-tumoral NK cell cytotoxicity. We demonstrate that induction of CCL5 by LCMV is specific for a subgroup of melanomas and that transgenic expression of CCL5 in a melanoma cell line enhances the responsiveness of LCMV therapy. This indicates that additional mechanisms influencing the responsiveness to LCMV therapy, exist.

In conclusion, we generated insight into the treatment efficiency of viro- and/or immunotherapy of human melanoma. As a similar correlation between CCL5, NK cells and tumor regression can exist in patients, we believe that LCMV-mediated immunotherapy is a new promising therapeutic approach in melanomas.

## Materials and Methods

### Viruses

The LCMV strain WE was kindly provided by Rolf Zinkernagel (Institute of Experimental Immunology, ETH, Zurich, Switzerland). LCMV was propagated in L929 cells, which were purchased from ATCC (CCL-1).

### Cell Lines

Human melanoma cells lines Ma-Mel-86a, Ma-Mel-86c and Ma-Mel-51 (36, 37), UKE-Mel-118b and UKE-Mel-118c were established from melanoma metastases after patient written informed consent and institutional review board approval. B16-Ova-CCL5 (50) were described previously.

### Mice

All animals were housed in single ventilated cages. Animal experiments were authorized by the Landesamt für Natur, Umwelt und Verbraucherschutz (LANUV) Nordrhein-Westfalen and in accordance with the German law for animal protection and/or according to institutional guidelines at the Ontario Cancer Institute of the University Health Network.

### Histological Analysis and Immunofluorescence

Histological analyses were done on snap frozen tissue harvested from tumor bearing mice. Briefy, sections were fixed with acetone for 10 min and non-specific antigens were blocked in PBS containing 2% FCS for 15 min. Staining for CCL5/RANTES was done using anti-RANTES (ab 9679; lot GR5419-63); VL-4 to stain LCMV or anti-NK1.1 (Cat 4322177 eBioscience). Antibodies were diluted 1:100. After 1 h of incubation, sections were washed with PBS containing 2% FCS and incubated for 1 h with secondary antibodies (dilution of 1:100). After mounting (S3023, Dako), images were acquired with a fluorescence microscope (KEYENCE BZ II analyser). Immunofluorescence was performed using cells grown on coverslips. Different human melanoma cells (Ma-Mel-86a, Ma-Mel-51 and Ma-Mel-86c) were grown (2 × 10^5^ cells per well in a 24 well plate) on cover slips. After fixation with 4% Formalin for 30 min, Triton X solution (1%) was added and incubated for 20 min room temperature for permeabilization. After washing, 10% FCS in PBS was added per well to block non-specific binding followed by a 1 h incubation. Primary antibodies were added and samples were incubated for 1 h at room temperature. After washing, secondary antibodies were added (1:100 dilution) followed by a 1 h incubation, washing and mounting (S3023, Dako). Images were acquired with a fluorescence microscope (KEYENCE BZ II analyser).

### Reverse Transcription and Quantitative RT-PCR

Total RNA was isolated by using TRIzol (Ambion), reverse transcribed into complementary DNA using Quantitect Reverse Transcription Kit (Qiagen) and analyzed by qRT-PCR using the SYBR Green Master Mix (Applied Biosystems, Darmstadt, Germany) or using TaqMan gene expression assay (Life Technologies) Relative gene expression levels were calculated using the ΔΔCt method.

### Tumor Induction

Patient derived melanoma cells were maintained at 37°C with 5% CO_2_ in DMEM medium supplemented with 10% heat-in-activated fetal calf serum (FCS), and 1% penicillin, streptomycin and glutamine. Cells were injected subcutaneously on the left flank of mice. Tumor size was determined by the formula (L×W×W)/2, with L = length, W = width, on the indicated days.

### Inhibitor Treatments

For the *In vivo* Maravoric treatment, Mice were treated twice a week with 50 mg/kg/mouse via oral gavage for a period of 3 weeks.

### Statistical Analysis

If not mentioned otherwise, data are expressed as the arithmetic mean ± SEM and n represents the number of mice or independent experiments. Student's *t*-test (two-tailed, if not indicated otherwise) in case of normal distribution or in case of multiple comparisons ANOVA, were used to detect statistically significant differences. *P*-values of 0.05 or less were considered statistically significant. Statistical analyses and graphical presentations were computed with Graph Pad Prism software (Graph Pad, La Jolla, USA).

## Data Availability Statement

All datasets generated for this study are included in the article/supplementary material.

## Ethics Statement

The animal study was reviewed and approved by Landesamt für Natur, Umwelt und Verbraucherschutz (LANUV)Nordrhein-Westfalen.

## Author Contributions

HB, GZ, DH, DS, HX, AP, MS, PL, and KL: conceptualization. HB, GZ, TH, JL, TA, RS, S-KF, MB, LC, FL, MA, FZ, VK, VD, TB, YM, MS, AB, HX, and BT: investigation. HB, GZ, TH, and JL: formal analysis. HB, GZ, JL, and KL: writing—original draft. HB, GZ, JL, JV, DH, AP, MS, AAP, PL, and KL: writing—review and editing. DH, AP, MS, PL, and KL: resources. DS, DH, AP, MS, PL, and KL: supervision. All authors contributed to the article and approved the submitted version.

## Conflict of Interest

JV, S-KF, MB, RS, AB, HX, PL, and KL declare that they are involved in the development of LCMV for clinical application in oncology in cooperation with or as employees of Abalos Therapeutics GmbH. The remaining authors declare that the research was conducted in the absence of any commercial or financial relationships that could be construed as a potential conflict of interest.
