# Supplementary figures and images for: Arenavirus Induced CCL5 Expression Causes NK Cell-Mediated Melanoma Regression
Source: Front Immunol. 2020 Aug 21;11:1849. doi: 10.3389/fimmu.2020.01849 (PMC7472885; doi:10.3389/fimmu.2020.01849)

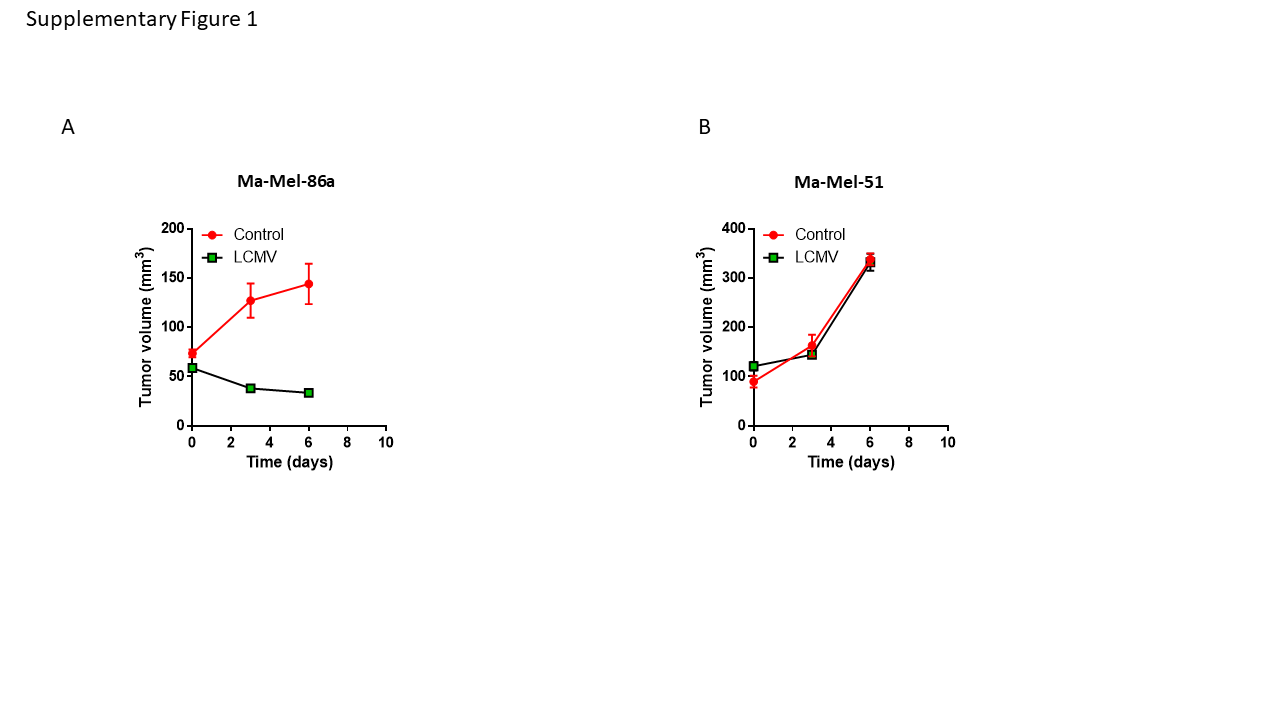

Supplement: Supplementary file 1 [file Image_1.tif]

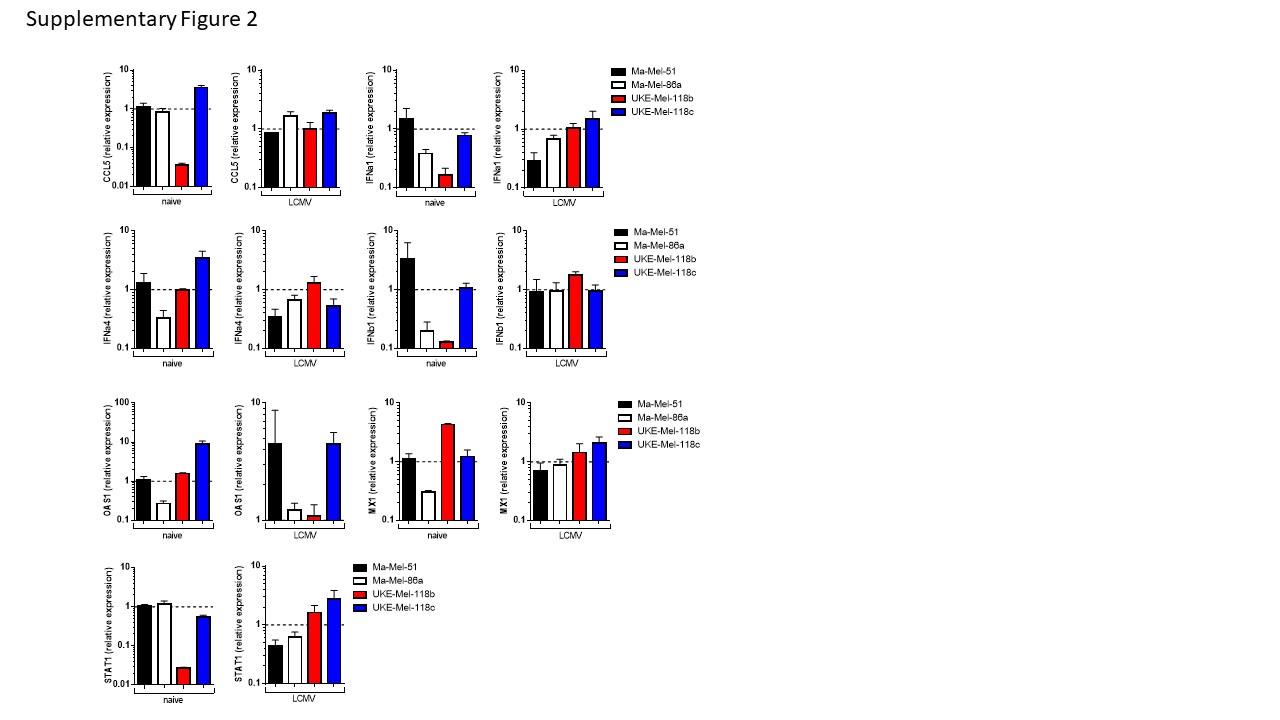

Supplement: Supplementary file 2 [file Image_2.tif]
